# Carcinosarcoma of the parotid gland with abdominal metastasis: a case report and review of literature

**DOI:** 10.1186/s12957-018-1406-6

**Published:** 2018-06-02

**Authors:** Chang Gok Woo, Seung-Myoung Son

**Affiliations:** Department of Pathology, Chungbuk National University Hosipital, 776, 1Sunhwan-ro, Seowon-gu, Cheongju, 28644 South Korea

**Keywords:** Carcinosarcoma, Parotid gland, Metastasis, Osteosarcoma, Abdominal cavity

## Abstract

**Background:**

Carcinosarcoma of the salivary gland is a rare aggressive malignant tumor, composed of a mixture of carcinomatous and sarcomatous components. The most common metastatic sites include the lungs, bones, and central nervous system.

**Case presentation:**

This report describes a rare case of carcinosarcoma of the parotid gland with an osteosarcoma as sarcomatous component in a 72-year-old man who had a history of low anterior resection for rectal cancer. Six months after parotidectomy, he presented abdominal pain as a symptom of abdominal metastasis by the sarcomatous component. At that time, the possibility of abdominal metastasis was overlooked because of the history of abdominal surgery. After several days of conservative treatment, emergency laparotomy was done. However, he died of acute respiratory distress syndrome.

**Conclusions:**

Awareness of the possibility of abdominal metastasis by salivary carcinosarcoma may help in managing patients with a history of abdominal surgery.

## Background

Carcinosarcoma, also called true malignant mixed tumor, is an aggressive malignant neoplasm of the salivary gland. It is an extremely rare malignancy, comprising 0.04 to 0.16% of all salivary gland tumors and 0.4% of all malignant salivary gland neoplasms. The mean age at presentation is in the sixth to seventh decade of life, but can range from 14 to 87 years [1–3]. Although de novo carcinosarcomas can occur in the salivary gland, many patients have a history of long standing or recurrent pleomorphic adenomas, a condition described as carcinosarcoma ex pleomorphic adenoma [4–6]. Most of these lesions arise in the major salivary glands, with two thirds in the parotid glands [2]. Patients typically present with a rapidly growing mass.

These tumors are composed of a mixture of distinct carcinomatous and sarcomatous components with either component capable of metastasis. Malignant epithelial components of salivary gland carcinosarcomas include squamous cell carcinoma and adenocarcinoma, whereas malignant mesenchymal components include chondrosarcoma, fibrosarcoma, leiomyosarcoma, osteosarcoma, and liposarcoma, in that order of frequency [1]. The most common metastatic sites include the lungs, bones, and central nervous system [2–4].

To date, 10 cases of carcinosarcoma of salivary glands with osteosarcomatous element have been published, of which only lung metastasis have been found [7–16]. We describe a case of carcinosarcoma arising in the parotid gland with an osteosarcomatous component which metastasized to abdominal cavity; to our knowledge, this is the first patient so described.

## Case presentation

**Fig. 1:**
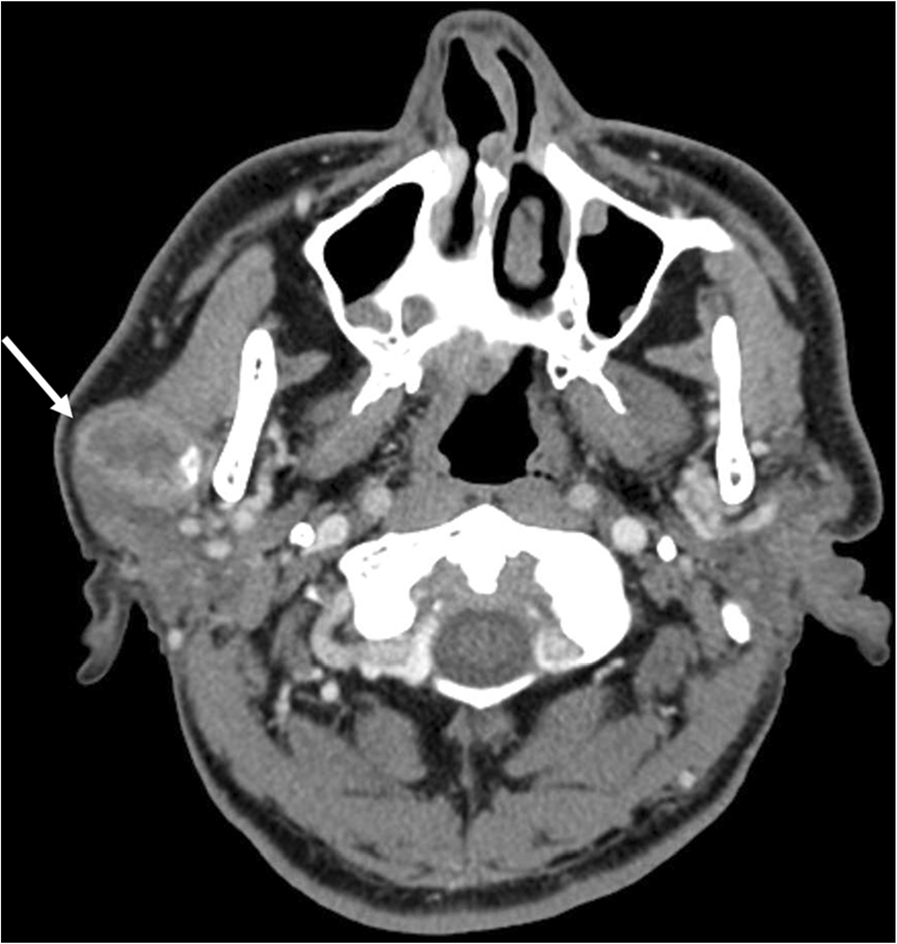
A heterogeneous enhancing solid lesion with calcification (arrow) was identified in the superficial lobe of right parotid gland on neck CT

A 72-year-old man presented with a mass in the right parotid area that had become rapidly enlarged for 2 months. His medical history included a subtotal gastrectomy for a gastric ulcer 20 years earlier and low anterior resection for moderately differentiated adenocarcinoma of the rectum (Stage T1N0) 1 year earlier. Preoperative computed tomography (CT) showed a relatively well-defined heterogeneous enhancing solid lesion with calcification in the superficial lobe of the right parotid gland, with no indications of metastasis to the regional lymph nodes (Fig. 1).

**Fig. 2:**
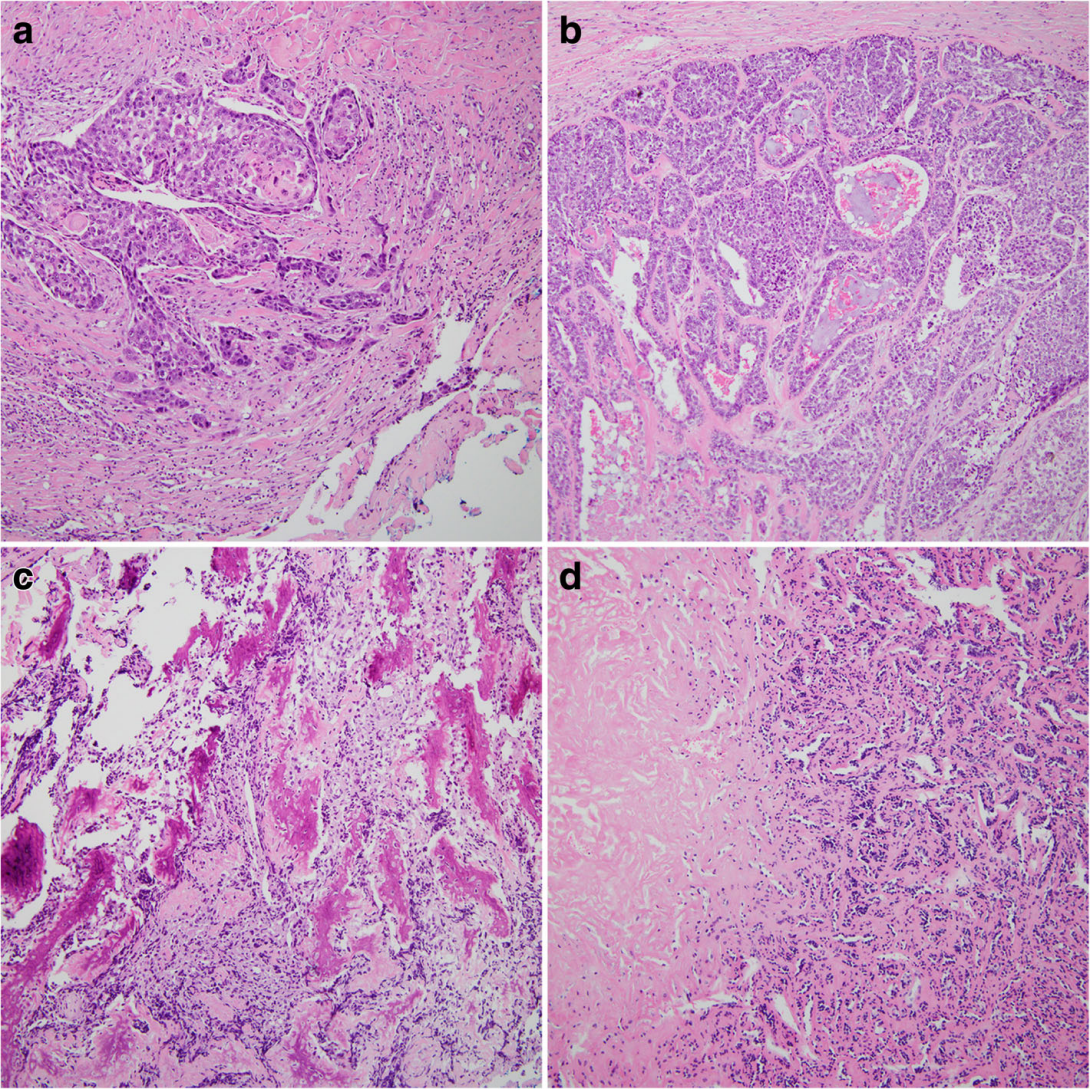
**a** The tumor cells showed features of squamous cell carcinoma, which were composed of abundant pink cytoplasm, moderate atypia, and focal keratinization (magnification, × 100). **b** The poorly differentiated adenocarcinoma components were consisted of solid nests of poorly differentiated tumor cells with ovoid nuclei and prominent nucleoli (magnification, × 100). **c** The sarcoma component consisted mainly of osteosarcoma, characterized by neoplastic bone and severe cellular anaplasia (magnification, × 100). **d** In pleomorphic adenoma component, the tubular and acinar structures formed by epithelial cells were compressed by abundant hyalinized eosinophilic matrix (magnification, × 100)

**Fig. 3:**
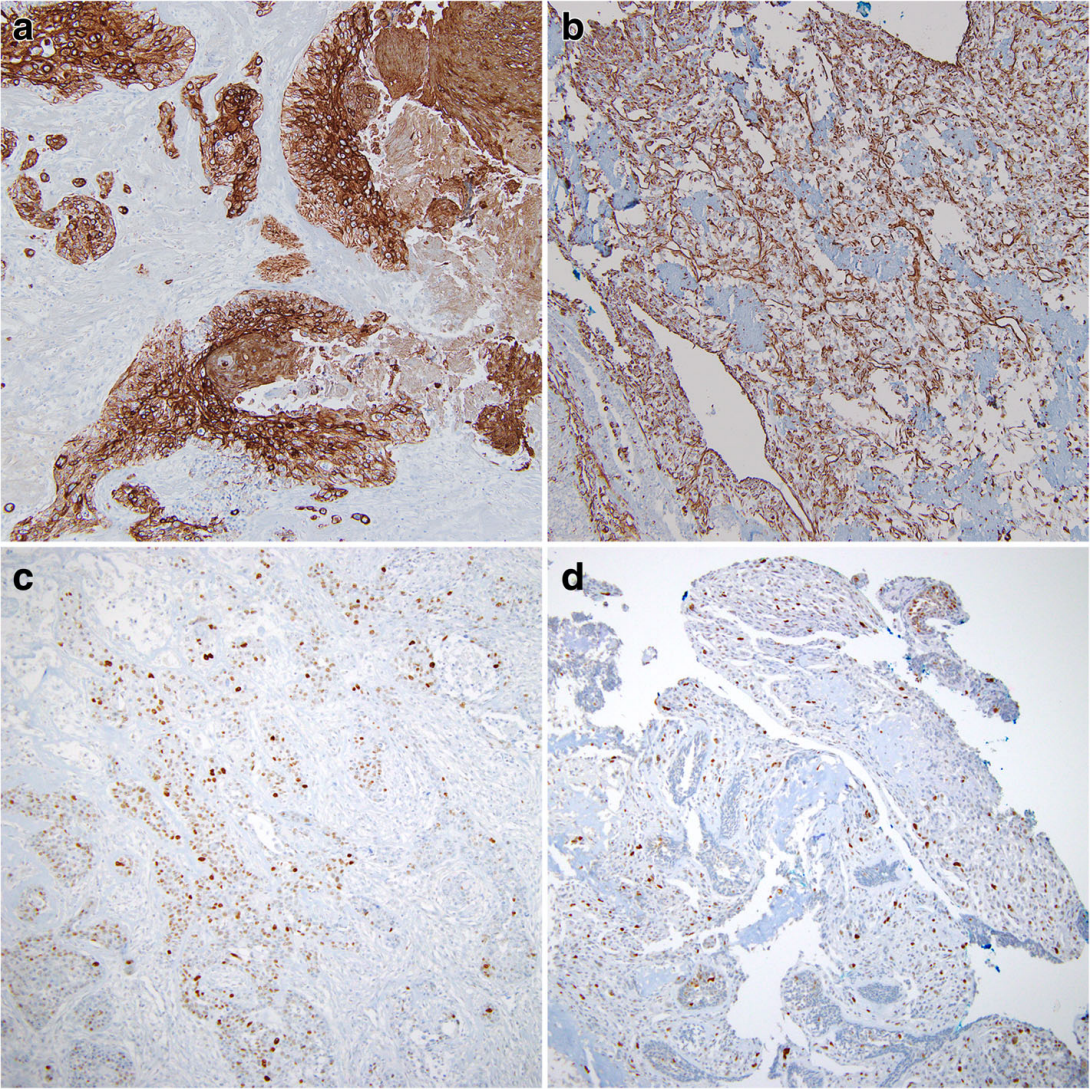
Immunohistochemistry showed that carcinomatous components were positive for cytokeratin AE1/AE3 (**a**) and sarcomatous components were positive for vimentin (**b**). Both components showed some degree of nuclear immunoreactivity for p53 (**c** carcinoma component; **d** sarcoma component) (magnification, all × 100)

Fine-needle aspiration specimen showed that the tumor contained many clusters of malignant epithelial cells and scattered atypical spindle cells on a necrotic background. Under suspicion of a malignant tumor, the patient underwent total parotidectomy. Gross examination of the specimen revealed a multifocal, ill-defined, grayish-white, and heterogeneous solid tumor, accompanied by calcification and measuring 3 × 2.5 cm. Microscopically, the tumor was composed of two malignant components, carcinoma (Fig. 2a, b) and sarcoma (Fig. 2c), with multifocal invasion (> 1.5 mm) of the capsule and adjacent tissues on a background of pre-existing pleomorphic adenoma (Fig. 2d). The carcinoma component of the tumor consisted of squamous cell carcinoma and poorly differentiated adenocarcinoma, whereas the sarcoma component consisted mainly of osteosarcoma, characterized by neoplastic bone and severe cellular anaplasia. Many mitotic figures and necrotic foci were observed. The external resection margin showed tumor involvement. Immunohistochemical staining showed that the carcinoma cells were positive for cytokeratin AE1/AE3 (Fig. 3a) and the osteosarcoma component was diffusely positive for vimentin (Fig. 3b). Both components showed some degree of nuclear immunoreactivity for p53 (Fig. 3c, d).

**Fig. 4:**
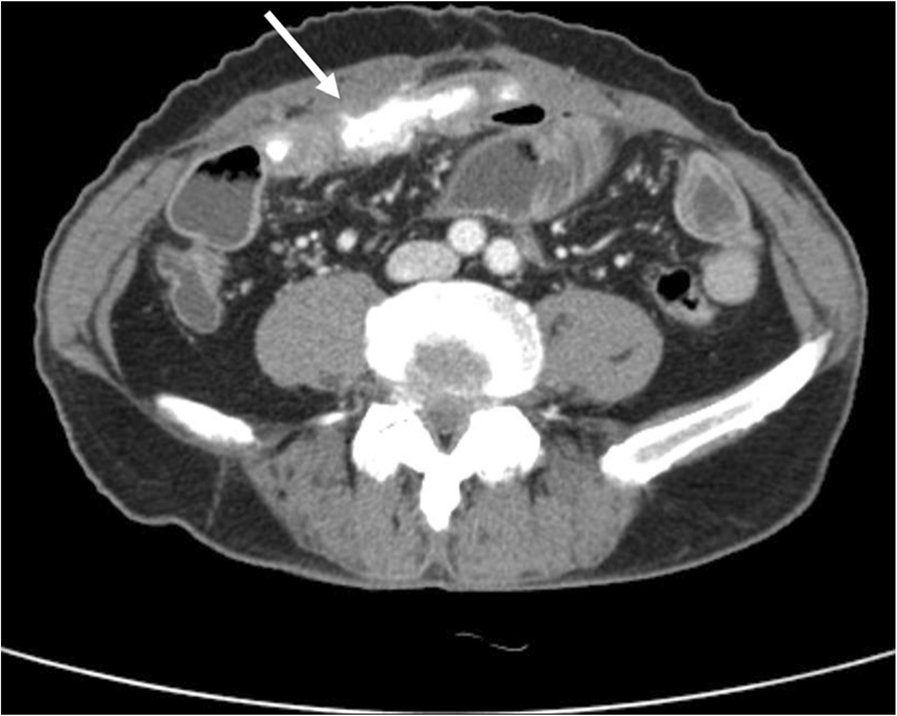
A localized high density material (arrow) in the abdominal cavity was presented in abdominal CT

**Fig. 5:**
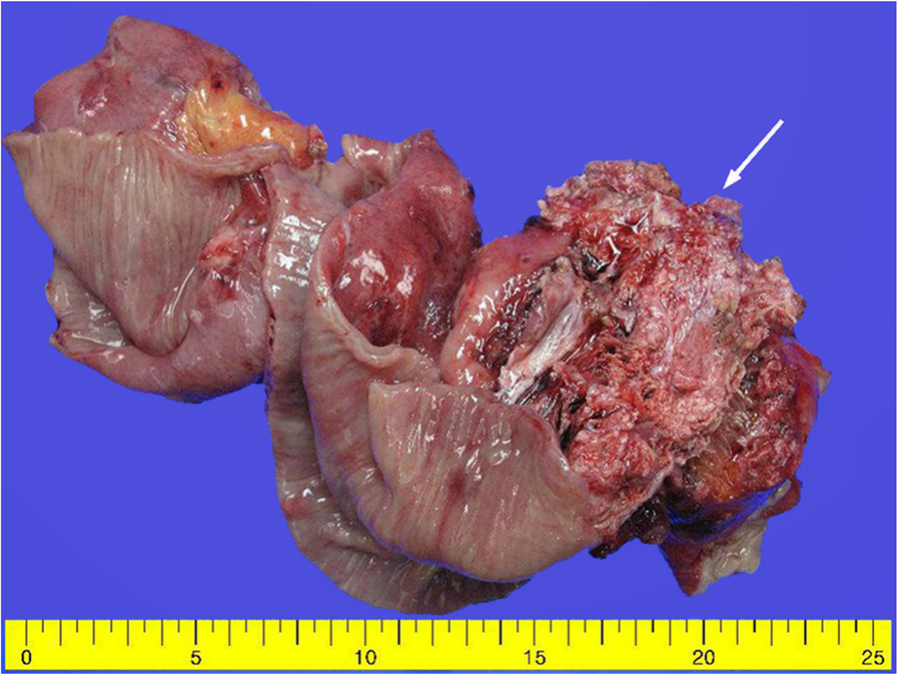
Bulky, gritty, and hemorrhagic masses (arrow) adhered to the small intestinal mesentery were observed

**Fig. 6:**
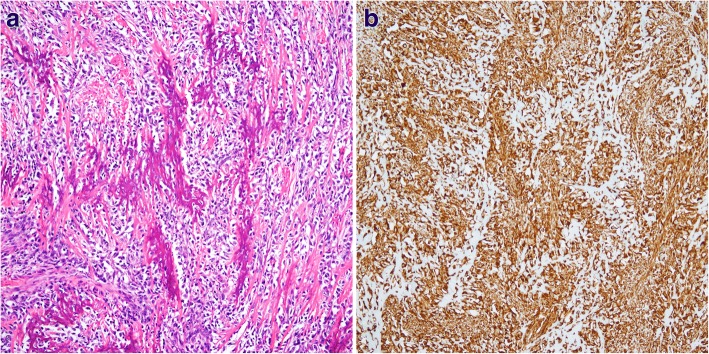
**a** Microscopically, the abdominal mass showed the osteosarcoma (magnification, × 100). **b** The immunostaining for vimentin was diffuse positive (magnification, × 100)

Following surgery, the patient received postoperative radiation therapy. Six months later, he complained of abdominal pain. Abdominal CT showed a distension of small bowel with luminal narrowing and localized high density material in the abdominal cavity (Fig. 4). The lesion was regarded as mechanical obstruction with postoperative adhesion because the patient had undergone previous abdominal surgery for rectal adenocarcinoma. After conservative management for several days, he experienced severe abdominal pain and an increased heart rate, and an emergency laparotomy was performed. Multiple hard calcified masses were observed in the abdominal walls, omentum, and mesentery, and mass excision with small bowel resection was done. On gross examination, bulky, gritty, and hemorrhagic mass adhered to the mesentery was identified (Fig. 5). Microscopically, the tumors were identified as osteogenic sarcomas, with histologic features identical to those of the osteosarcomatous component of the carcinosarcoma of the parotid gland (Fig. 6a). The tumor cells were positive for vimentin on immunohistochemical staining (Fig. 6b). Because of its rapid development over 6 months and no history of osteosarcoma at any sites, we concluded that the abdominal osteosarcoma was metastatic from the carcinosarcoma of the parotid gland. The patient was postoperatively admitted to the intensive care unit and died of acute respiratory distress syndrome (ARDS) caused by aspiration pneumonia.

## Discussion and conclusions

Malignant mixed tumors of the salivary gland can describe three distinct histologic entities: carcinoma ex pleomorphic adenoma (the most common), metastasizing mixed tumor, and carcinosarcoma [1]. More than 99% of these tumors arise from pleomorphic adenomas, with only 0.2% being primary carcinosarcomas or true malignant mixed tumors [17]. Two hypotheses have been advanced to explain the histogenesis of carcinosarcoma. The convergence hypothesis suggests that these tumors are multiclonal and are derived from two or more types of mesenchymal and epithelial stem cells. The divergence hypothesis suggests that these tumors are monoclonal, with a single totipotent stem cell differentiating into epithelial and mesenchymal cells [18].

Surgery followed by radiation therapy may aid in the treatment of salivary carcinosarcoma. Despite treatment, over half of these patients die of local recurrence and/or metastasis and the average survival of these patients is 3.6 years [2, 7].

Only 10 cases of carcinosarcoma with osteosarcoma as the sarcomatous component have been reported. The clinicopathological features and outcomes of the reported cases are detailed in Table 1. Our reviews exhibited a male predominance (72.7%) with an age range of 35 to 83 years (median, 64 years). All cases had an adenocarcinoma as the carcinomatous component, and three cases also showed squamous cell carcinoma elements. The sarcomatous components other than osteosarcoma were chondrosarcomas, rhabdomyosarcomas, and fibrosarcomas. Of the 11 cases, 8 cases occurred in the parotid gland. All patients underwent surgical resection and 6 of them received radiotherapy and/or chemotherapy. Distant metastasis occurred in five cases, of which four cases involved the lung. In the present case, the sarcomatous component was an osteosarcoma, and the carcinomatous components were adenocarcinoma and squamous cell carcinoma. The tumor metastasized to the abdominal cavity by the osteosarcomatous component alone, which has not been reported to date.

**Table 1:** Review of previously reported cases of carcinosarcoma of the salivary glands with osteosarcoma components

Reference	Age (years)/sex	Location	Histologic type of carcinoma	Histologic type of sarcoma	Metastasis/site	Histologic type of metastatic tumor	Treatment	Outcome
Garner et al. 1989 [7]	57/F	Parotid gland	Adenocarcinoma	Osteosarcoma, chondrosarcoma	No	(−)	Left parotidectomy and radical neck dissection	Local recurrence at 6 weeks and died
Yamashita et al. 1990 [8]	52/M	Submandibular gland	Adenocarcinoma, squamous cell carcinoma	Osteosarcoma, chondrosarcoma, fibrosarcoma	No	(−)	Excision and right modified neck dissection with chemotherapy	No recurrence for 5 months
Bleiweiss et al. 1992 [9]	64/M	Submandibular gland	Adenocarcinoma	Osteosarcoma	No	(−)	Partial mandibulectomy with radical neck dissection	Local recurrence at 4 months
de la Torre et al. 1995 [10]	83/F	Parotid gland	Adenocarcinoma	Osteosarcoma, chondrosarcoma	Yes/lung	NR	Wide excision	Local recurrence and lung metastasis, died after 1 year
Carson et al. 1995 [11]	51/F	Parotid gland	Adenocarcinoma	Osteosarcoma	No	(−)	Total parotidectomy with chemotherapy	Local recurrence at 7 months and died at 9 months
Gogas et al. 1999 [12]	77/M	Submandibular gland	Salivary duct carcinoma	Osteosarcoma. chondrosarcoma, rhabdomyosarcoma	Yes/lung	NR	Wide excision with radiotherapy and chemotherapy	Lung metastasis at 3 months
Sironi et al. 2000 [13]	77/M	Parotid gland	Squamous cell carcinoma, adenocarcinoma,	Osteosarcoma	Yes/NR	NR	Right total parotidectomy with radical neck dissection	Metastasis at 3 months
Mardi et al. 2004 [14]	59/M	Parotid gland	Adenocarcinoma	Osteosarcoma, chondrosarcoma	NA	NA	NA	NA
Jang et al. 2011 [15]	67/M	Parotid gland	Adenocarcinoma	Osteosarcoma	Yes/lung	Osteosarcoma	Left parotidectomy with radiotherapy	Lung metastasis at 5 months
Jha et al.2017 [16]	35/M	Parotid gland	Adenocarcinoma	Osteosarcoma	No	(−)	Total radical parotidectomy with radiotherapy	No recurrence for 12 months
Present case	72/M	Parotid gland	Adenocarcinoma, squamous cell carcinoma	Osteosarcoma	Yes/abdominal cavity	Osteosarcoma	Total parotidectomy with radiotherapy	Metastasis at 6 months and died

NA, not available, *NR* not reported

The present patient underwent surgery and adjuvant radiotherapy. After 6 months, he complained of abdominal pain. At that time, the signs of small bowel obstruction with high density material in the abdominal cavity found on CT were regarded as an effect of his abdominal surgery for rectal adenocarcinoma 1 year earlier. Therefore, the management was only conservative, without surgery, and the possibility of metastasis from the salivary malignancy was overlooked. Later, rapid growth of the metastatic tumor resulted in an emergency laparotomy. Abdominal metastases of the mesentery, omentum, and peritoneum were observed. The patient experienced postoperative complications and died of ARDS.

We described a case of salivary gland carcinosarcoma harboring an osteosarcomatous component with abdominal metastasis consisted entirely of the sarcomatous element. Awareness of the possibility of abdominal metastasis by the sarcomatous component of salivary carcinosarcoma may help in managing patients with a history of abdominal surgery.

## Abbreviations

CT: Computed tomography
ARDS: Acute respiratory distress syndrome
